# The oblique approach for US-guided peripheral venous cannulation in phantom models

**DOI:** 10.1186/2036-7902-6-S1-A15

**Published:** 2014-01-31

**Authors:** DE Morato, S Terp, M Chilstrom, CN Lam, M Menchine, T Mailhot

**Affiliations:** 1 Department of Emergency Medicine, LAC+USC Medical Center, Los Angeles, CA, USA

## Objective

Ultrasound (US) guided peripheral vascular access is a valuable skill for physicians. A novel approach to vascular cannulation called the oblique technique has been described, which purports to eliminate the disadvantages of the traditional techniques (short axis, SA; and long axis, LA) while preserving their advantages. This technique has not been well studied. We aimed to compare the three techniques to determine which is associated with the highest rate and fastest time to cannulation, and lowest rate of posterior vessel wall puncture (PVWP). We hypothesized that the oblique technique would be superior to the others.

## Methods

Sixty subjects including 35 Emergency Medicine residents and 25 senior medical students volunteered to participate. All subjects received standardized instruction on how to perform the three techniques prior to the study. Their order of approach (SA, LA, or oblique) was randomized, and subjects were timed as they attempted to cannulate disposable peripheral vascular phantoms. Following the attempts, the phantoms were deconstructed to assess outcomes.

## Results

There was a statistically significant reduction in the rate of cannulation failure with the oblique technique (12%, 95% CI 3-20%) versus SA (22%, 95% CI 11-32%) or LA (32%, 95% CI 20-44%) (Pearson chi2 7.1; df=2; p=0.03). Mean time to cannulation (in seconds) was not observed to be significantly different between the SA (31, 95% CI 24-37), LA (28, 95% CI 23-33), and oblique (34, 95% CI 25-44) techniques. There was no significant difference in the rate of PVWP between the SA (18%, 95% CI 8-28%), LA (22%, 95% CI 11-32%), and oblique (15%, 95% CI 6-24%) techniques.

## Conclusion

The type of US-guided technique employed for peripheral vascular access was significantly associated with success of cannulation in this study. The oblique technique was associated with a near 2 and 3-fold decrease in failure rate compared with the SA and LA techniques, respectively. The three techniques for US-guided peripheral vessel cannulation are equivalent with respect to time to cannulation and rates of PVWP

